# Duodenal Stenosis With Significant Edema Due to Complication From an Enteral Nutrition Tube: A Case Report

**DOI:** 10.7759/cureus.83749

**Published:** 2025-05-08

**Authors:** Shinichi Ijuin, Aika Yano, Yukihide Nakatani, Haruki Kaneda, Satoshi Ishihara

**Affiliations:** 1 Emergency and Critical Care Medicine, Hyogo Emergency Medical Center, Kobe, JPN

**Keywords:** duodenal bulb, duodenal edema, duodenal stenosis, enteral nutrition, enteral nutrition tube

## Abstract

Enteral nutrition (EN) is a well-established feeding method for individuals with dysphagia or those with intubation. Although EN tube placement is generally well tolerated, complications can sometimes occur. A 60-year-old male was brought to our hospital after being struck by a container that had slid off the trailer bed. He sustained severe traumatic brain injury, pelvic fracture, and open fracture of the lower leg, for which appropriate treatment was initiated. No abnormalities were identified in other organs on CT. As mechanical ventilation management was continued, EN was administered via an EN tube placed in the duodenal bulb. On day 26, he experienced excessive vomiting and an increase in gastric residual volume (GRV). Despite medication adjustments and reduced feeding volume, only minimal improvement was observed. On day 33, upper gastrointestinal endoscopy revealed significant edematous changes in the duodenal bulb, near the tip of the EN tube, indicating a possible stenosis. The EN tube was repositioned into the stomach, and he was managed with fasting and fluid replacement, leading to a gradual reduction in GRV. As illustrated in this report, the placement of an EN tube in the duodenal bulb - an area located at an acute angle to the descending portion of the duodenum and part of the retroperitoneal space - should be avoided to prevent potential obstruction.

## Introduction

Enteral nutrition (EN) is a routinely performed procedure worldwide in individuals with dysphagia or those requiring intubation [1]. An EN tube is a relatively safe and effective method for delivering food, water, and medications directly into the lumen of the stomach or small intestine. Gastroparesis often occurs in critically ill patients [2], potentially leading to aspiration due to delayed gastric emptying and vomiting [3]. Therefore, post-pyloric EN has emerged as an alternative method for gastrointestinal feeding in critically ill patients. This method is expected to deliver nutrients directly to the intestines beyond the duodenum, helping to ensure more consistent absorption while minimizing the risk of aspiration and vomiting [4,5].

Although EN tube placement is usually well tolerated, complications such as bleeding, esophageal or gastrointestinal perforation, ulceration, and inadvertent removal of the tube, etc., can sometimes occur, including post-pyloric EN [4,6-8]. However, to the best of our knowledge, no previous reports have described duodenal stenosis caused by an EN tube. We report a rare, complex case of duodenal stenosis resulting from duodenal wall edema due to mechanical contact with an EN tube tip.

## Case presentation

A 60-year-old male was brought to our institute after sustaining injury, having been struck by a container that had slid off the trailer bed. When the prehospital emergency team had arrived at the scene five minutes after the crash, the patient had been on the back of the motorcycle, but he had not suffered any abdominal injuries. After intubation by an emergency medical service, he had been transferred to our hospital. On arrival at the trauma bay, the patient’s blood pressure level stabilized at 110/65 mmHg, and his heart rate was 134 bpm, indicating unstable hemodynamics. His Glasgow Coma Scale (GCS) score was 6 (E1, VT, M4). Consequently, fluid resuscitation with blood transfusion was performed. When the hemodynamic state gradually improved, a contrast media-enhanced CT scan was conducted, revealing traumatic brain injury (subdural hematoma, subarachnoid hemorrhage, and skull fracture), pneumothorax, pelvic fracture, and open fracture of the lower leg. No abnormalities were identified in other organs by CT scan, including the duodenum. Subsequently, a craniotomy for traumatic brain injury and external fixation for lower leg fracture were performed. After the surgery, he was admitted to the ICU.

**Figure 1:**
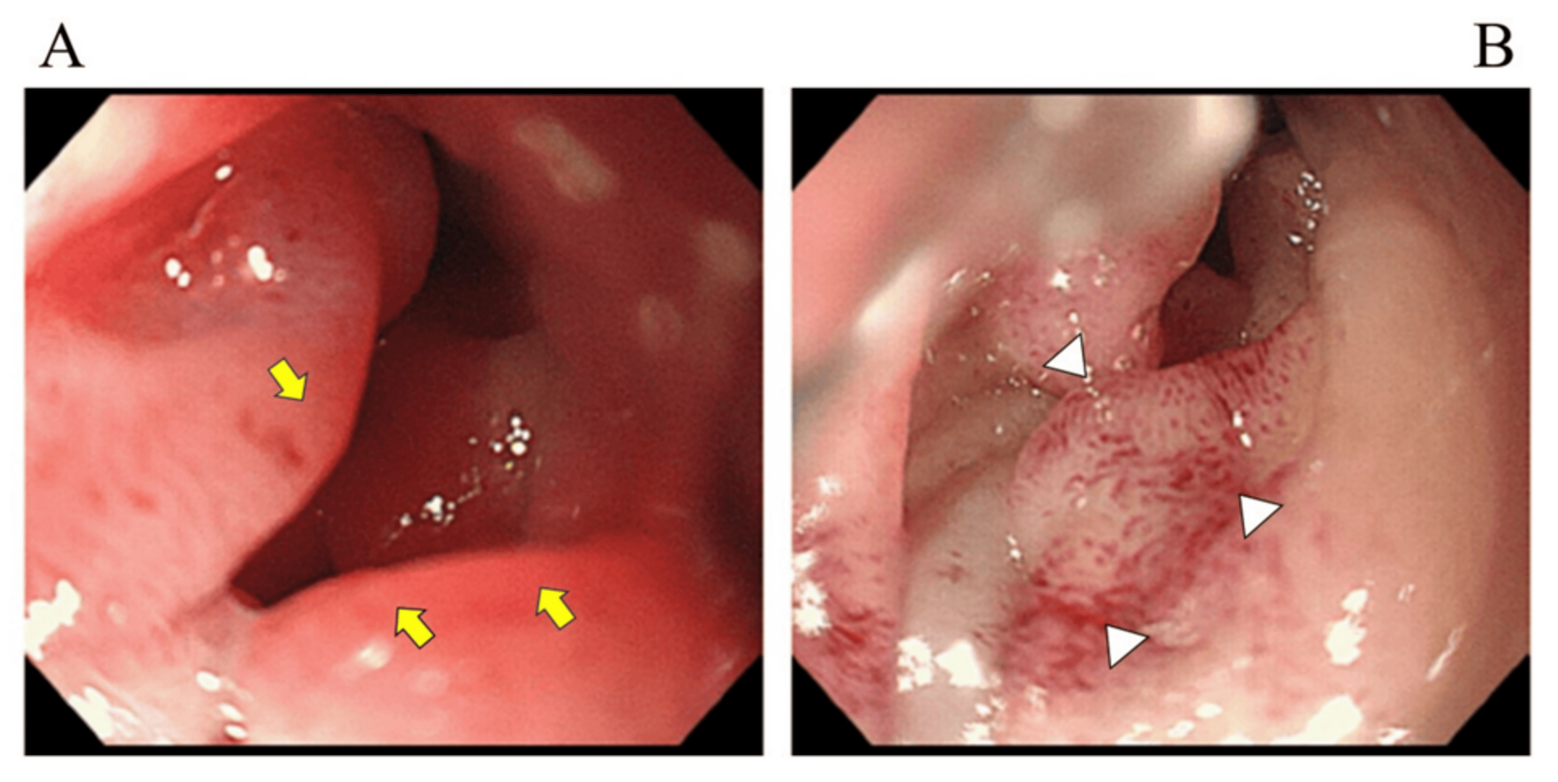
Upper gastrointestinal endoscopy in the duodenal bulb on day 33 A: Significant edematous changes (arrows). B: Submucosal petechia (triangles)

Since mechanical ventilation management was continued, EN was initiated on day two using an oligomeric formula via an EN tube (New Enteral Feeding Tube; CardinalHealthTM, Tokyo, Japan). Given the severity of his traumatic brain injury, we tried post-pyloric feeding into the small intestine under fluoroscopy guidance to mitigate the risk of vomiting caused by gastroparesis. However, as bypassing the duodenal bulb was difficult, an EN tube tip was placed in the duodenal bulb, hoping that it would advance to the distal portion by gastrointestinal peristalsis. After starting EN, the EN volume was increased gradually without trouble, and the postoperative course was also judged to be favorable, including CT findings on day 20. However, on day 26, the patient experienced excessive vomiting along with an increase in gastric residual volume (GRV). Despite adjustments to medication and a reduction in feeding volume, only minimal improvement was observed. Therefore, an upper gastrointestinal endoscopy was performed on day 33. The finding revealed significant edematous changes and submucosal bleeding spots primarily in the duodenal bulb, near the EN tube tip, indicating passage obstruction in this area (Figure 1).

**Figure 2:**
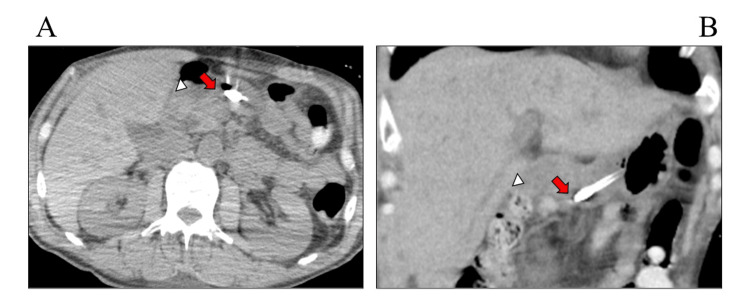
CT on day 20 revealed the tip of the enteral nutrition tube in the duodenal bulb (arrow) A: Axial scan. B: Coronal scan. Triangles show the transverse colon CT: computed tomography

As the endoscopy could not advance beyond the duodenal bulb, further findings could not be confirmed. During the endoscopy, we retrospectively checked the CT scan on day 20 again, and it was recognized for the first time that the EN tube tip was placed in the duodenal bulb (Figure 2).

**Figure 3:**
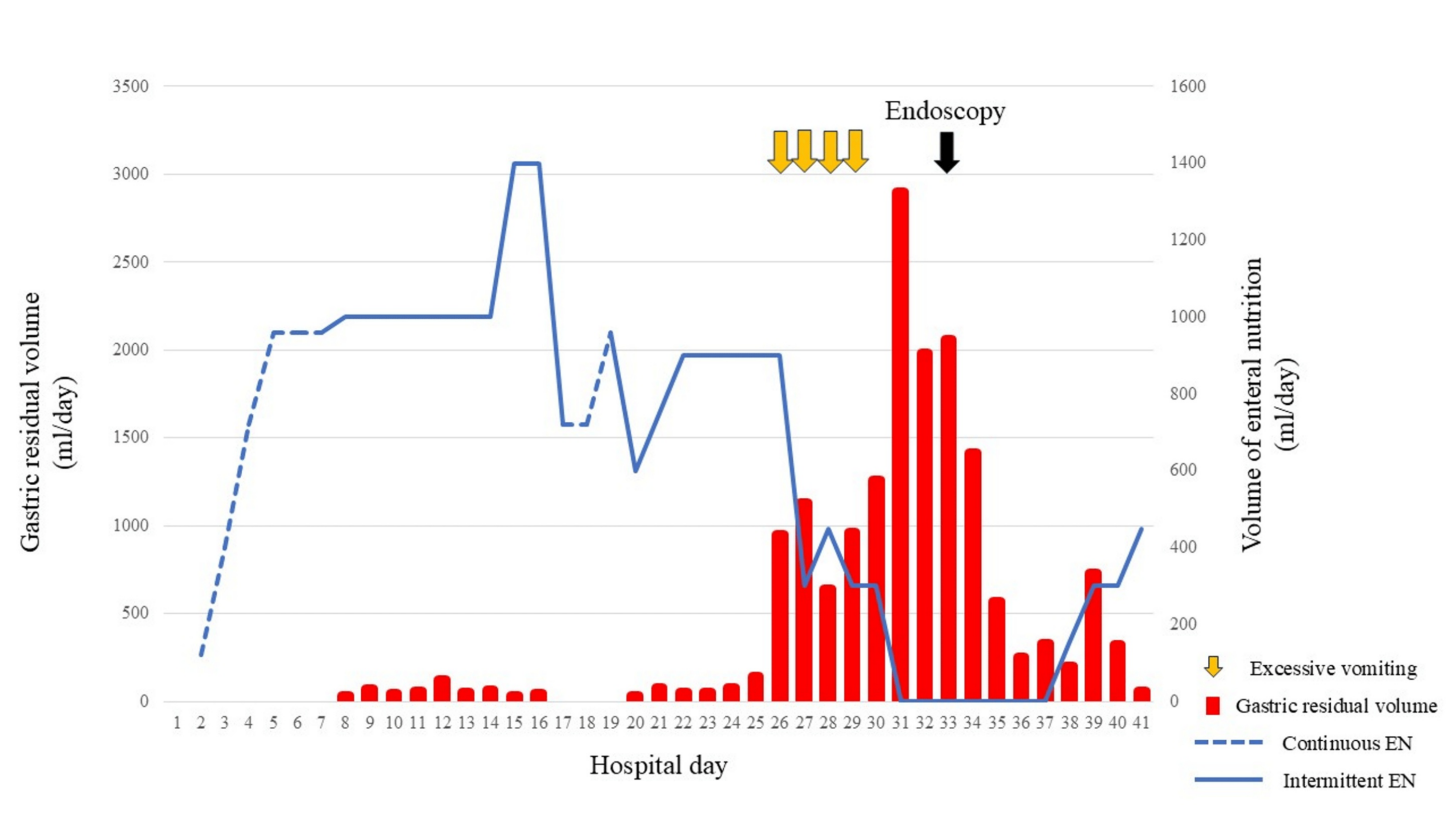
Clinical course of the volume of EN and gastric residual volume EN: enteral nutrition

The EN tube was drawn into the EN, and volume was increased gradually with the stomach, and the patient was observed with fasting and fluid replacement, leading to a gradual decrease in GRV (Figure 3). After repositioning the EN tube, his clinical course was uneventful, allowing EN to resume on day 37 according to the decrease in GRV. 

The patient was then transferred to a rehabilitation hospital on day 41, with a GCS score of 4 [9], before the second endoscopy.

## Discussion

**Figure 4:**
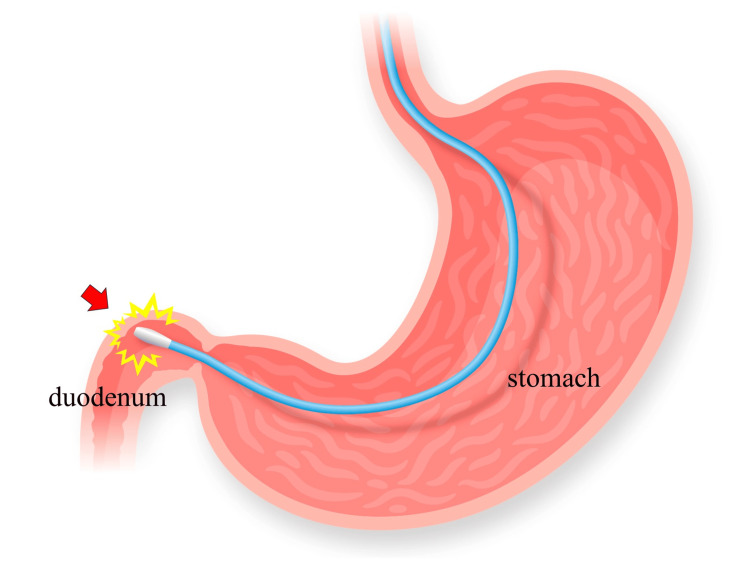
Schema of the mechanical contact of the tip of the enteral nutrition tube with the wall of the duodenal bulb (arrow) Image created by the authors

We have presented a case of significant edema caused by mechanical contact between the EN tube tip and the wall of the duodenal bulb. Given the severity of traumatic brain injury and weakened gastroparesis of the patient, post-pyloric EN was considered. However, because bypassing the duodenal bulb was technically difficult, intragastric placement was initially considered. Though we fully recognized that the post-pyloric EN is the best practice in such cases, an EN tube tip was placed in the duodenal bulb with a bent position within the stomach, hoping that the tube tip would advance toward the post-pyloric area by peristalsis, taking into account the risk of vomiting due to weaned gastrointestinal function by brain trauma injury [10,11]. Since the patient was transferred before the second endoscopy, the changes in the endoscopic findings could not be confirmed; however, we assume that this complication was due to the mechanical contact of the EN tube tip with the wall of the duodenal bulb (Figure 4). In addition, although the exact timing of the edematous change caused by the advancement of the EN tube is unclear, the CT scan on day 20 revealed the EN tube tip positioned in the duodenal bulb, suggesting that changes had likely occurred around that time.

An EN tube usually causes enteritis and stenosis of the passage because of edema. Several similar cases, such as gastrointestinal obstruction, have been reported [12-14]. This complication is typically due to the migration of the gastrostomy tube into the pyloric channel or the duodenal bulb, causing gastric outlet obstruction. The migration of the gastrostomy tube occurs as the duodenal bulb has an acute angle to the descending portion. Additionally, as the duodenum is a retroperitoneal organ with limited mobility, an EN tube tip may be more likely to come in contact with the intestinal wall.

However, to our knowledge, passage obstruction by secondary significant edema resulting from the placement of an EN tube has not been reported. Therefore, there appear to be no previously published cases reporting duodenal stenosis secondary to edema induced by an EN tube in this manner. Consequently, in addition to determining the appropriate position, such as intra-stomach or small intestine, fixing the EN tube tip to the duodenal bulb should also be avoided.

## Conclusions

We discussed a unique case of significant edema caused by mechanical contact between the EN tube tip and the wall of the duodenal bulb. When using an EN tube, physicians should be mindful of the position of the tube tip and recognize that intestinal stenosis, although rare, can occur as a late complication of tube placement. Therefore, an EN tube tip should not be fixed to the duodenal bulb, as this region forms an acute angle with the descending portion of the duodenum.
